# Using an ATR-FTIR Technique to Detect Pathogens in Patients with Urinary Tract Infections: A Pilot Study

**DOI:** 10.3390/s22103638

**Published:** 2022-05-10

**Authors:** Sheng-Wei Pan, Hsiao-Chi Lu, Jen-Iu Lo, Li-Ing Ho, Ton-Rong Tseng, Mei-Lin Ho, Bing-Ming Cheng

**Affiliations:** 1 Department of Chest Medicine, Taipei Veterans General Hospital, Taipei 11217, Taiwan; swpan2@vghtpe.gov.tw (S.-W.P.); liho@vghtpe.gov.tw (L.-I.H.); 2 School of Medicine, National Yang Ming Chiao Tung University, Taipei 12304, Taiwan; 3 Division of Pulmonary, Critical Care and Sleep Medicine, Department of Medicine, University of California San Diego, La Jolla, CA 92093, USA; 4 Department of Medical Research, Hualien Tzu Chi Hospital, Buddhist Tzu Chi Medical Foundation, No. 707, Sec. 3, Chung-Yang Rd., Hualien City 97002, Taiwan; hchopelu@gmail.com (H.-C.L.); jeniuluo@gmail.com (J.-I.L.); 5 Mastek Technologies, Inc., 4F-4, No. 13, Wuquan 1st Rd., Xinzhuang, New Taipei City 24892, Taiwan; simon@mastek-tw.com; 6 Department of Chemistry, Soochow University, No. 70, LinShih Rd., Shih-Lin, Taipei 11102, Taiwan; 7 Office of Research and Development, Tzu Chi University of Science and Technology, No. 880, Sec. 2, Chien-kuo Rd., Hualien City 97005, Taiwan

**Keywords:** urinary tract infections (UTIs), attenuated total reflection–Fourier-transform infrared spectroscopy (ATR-FTIR), pathogen, catheter-associated urinary tract infection (CAUTI)

## Abstract

Urinary tract infections (UTIs) are a leading hospital-acquired infection. Although timely detection of causative pathogens of UTIs is important, rapid and accurate measures assisting UTI diagnosis and bacterial determination are poorly developed. By reading infrared spectra of urine samples, Fourier-transform infrared spectroscopy (FTIR) may help detect urine compounds, but its role in UTI diagnosis remains uncertain. In this pilot study, we proposed a characterization method in attenuated total reflection (ATR)-FTIR spectra to evaluate urine samples and assessed the correlation between ATR-FTIR patterns, UTI diagnosis, and causative pathogens. We enrolled patients with a catheter-associated UTI in a subacute-care unit and non-UTI controls (total *n* = 18), and used urine culture to confirm the causative pathogens of the UTIs. In the ATR-FTIR analysis, the spectral variation between the UTI group and non-UTI, as well as that between various pathogens, was found in a range of 1800–900 cm^−1^, referring to the presence of specific constituents of the bacterial cell wall. The results indicated that the relative ratios between different area zones of vibration, as well as multivariate analysis, can be used as a clue to discriminate between UTI and non-UTI, as well as different causative pathogens of UTIs. This warrants a further large-scale study to validate the findings of this pilot research.

## 1. Introduction

Urinary tract infections (UTIs) are common diseases that affect the urinary system. More than 7 million office visits, 1 million emergency department visits, and over 100,000 hospitalizations are estimated to occur yearly in the United States, resulting in more than USD 33 billion in healthcare costs [1]. As a point-of-care test (POCT), a urine dipstick provides an indirect method for rapid UTI screening, but this test can yield false-negative or false-positive results in dilute urine samples [2,3]. To provide supportive evidence of a UTI, diagnostic technologies, including culture-based devices, enzymatic assays, and semiautomated urine analyzers, have been expanded with accuracies greater than that of simpler dipsticks [4].

Pathogen identification is crucial for the treatment of a given infectious disease. The current standard diagnostic methods for UTIs are urine microscopic examination, urine culture, and antibiotic susceptibility analysis; the latter two are pathogen-specific tests. However, these culture-based methods take at least 72 h; urine culture is not always performed in primary care settings and emergency departments [2,3,4]. In the absence of a rapid and accurate test to determine the pathogens of a UTI, current clinical guidelines advocate empirical antibiotic therapy [4], but inappropriate and prolonged empirical antibiotic use leads to the emergence of drug-resistant pathogens and adverse effects [5]. For rapid diagnosis, culture-independent techniques, such as polymerase chain reaction (PCR) and checkerboard DNA-DNA hybridization, have been developed [6]; however, time-consuming, costly, and labor-intensive issues remain unresolved [7,8]. There is hence a necessity to develop a more rapid and directly pathogen-oriented method to detect UTIs and pathogens. Several methods being considered at present are Raman spectroscopy, oligonucleotide-based molecular beacons, MALDI-TOF, plasmonics, electronic nose, flow cytometry, and nucleic acid-based diagnostics, which represent attractive molecular diagnostic modalities for a clinic [9,10,11,12,13,14,15,16,17,18,19,20,21,22,23,24]. The advantages of ATR-FTIR spectroscopy compared to other methods are the high signal-to-noise ratio, reduced dispersion, good spatial resolution, nondestructiveness, lack of sample preparation (or minimal preparation), low relative cost, and automated analysis [25,26]. On the other hand, the ATR-FTIR method can provide the fingerprint information of the targets and can be more versatile in detecting different pathogens in parallel in comparison to the ATR-localized surface plasmon resonance technique, which is based on the refractive index changes due to molecular binding [27]. 

In FTIR spectroscopy, conventional infrared spectra involve light in a wavelength range of 2500–20,000 nm, corresponding to wavenumbers of about ~4000–500 cm^−1^, which is generally called the mid-IR region. The energy of mid-IR photons matches the energy of molecular vibrational transitions, which are associated with the chemical and structural information of a sample. Due to their potential as a diagnostic tool for various diseases, infrared vibrational spectra have in recent years attracted increasing attention for tissues, cells, and biofluids, such as blood plasma or serum, urine, bile, ascitic fluid, and cerebrospinal fluid for cancer tests. In investigations of these kinds, urine has been studied with IR spectra; methods have been developed to detect and quantitate specific urinary components from IR spectra [28]. 

In clinical practice, a UTI can be classified as a complicated or uncomplicated form. Uncomplicated UTIs are seen mostly in females of all ages, but also in infant boys and older adult men [29]. In contrast, complicated UTIs occur in males and pregnant females as a result of the presence of factors that compromise urodynamics or host defenses [30]. Notably, indwelling urinary catheterization is the most common risk factor for a complicated UTI, namely catheter-associated urinary tract infection (CAUTI) [29]. Patients at risk of CAUTI are typically bedridden and have several comorbidities, and for whom rapid diagnosis of UTIs and accurate pathogen detection are important, as various bacterial and fungal infections can occur [31]. Steenbeke et al. reported that it was possible to use FTIR spectra to analyze urine sediments to differentiate causative bacteria in patients with a UTI [32]. It is essential, however, to investigate whether FTIR spectra are useful for detecting UTI, especially CAUTI, and to determine pathogens including nonbacterial fungal infections. 

In this work, as a pilot study, we aimed to propose a characterization method in attenuated total reflection–Fourier-transform infrared (ATR-FTIR) spectra to assess: (1) whether a tested urine sample was really an infected one that was obtained from a patient with a UTI; and (2) whether the species of pathogen existed in the infected urine samples. We tested urine samples directly from patients with indwelling urinary catheters at a subacute-care unit and healthy participants, and found that the ATR-FTIR patterns were correlated with pathogens in patients with UTIs. We noted the specific bands in the IR spectra, which were associated with molecular transitions as the markers, and the relative ratio between different area zones of vibration, as well as multivariate analysis, in the range of 1250–950 cm^−1^. These findings in the ATR-FTIR spectra may have value in helping to discriminate UTIs from non-UTIs, as well as the different causative pathogens of UTIs. 

## 2. Methods

### 2.1. Clinical Setting and Urine Culture

Healthy adult volunteers and inpatients with indwelling urinary bladder catheters in the respiratory care center at Taipei Veterans General Hospital (Taiwan) were enrolled in this study. From September 2018 to February 2021, 10 mL urine samples were collected from each participant after signing an informed consent (the institutional review board of this hospital approved the protocol under Nos. 2017-12-002CC and 2018-05-006CC). Simultaneously, in additional to the FTIR test, a routine urinalysis was performed immediately for each urine sample to check if there was supporting evidence of a urinary tract infection (UTI) or not. In brief, the diagnosis of UTI was confirmed by two independent physicians (S.-W.P. and L.-I.H.) to check if a participant had evidence of pyuria (urine leukocyte count ≥ 10/HPF) plus bacteriuria in a microscopic examination, as well as urine that was culture-positive for bacteria (≥10^3^ colony-forming units/mL of ≥1 bacterial species) plus compatible urinary symptoms and/or fever, in a manner previously described [33]. By definition, since all our inpatients had indwelling bladder catheters upon enrollment, a diagnosis of UTI made in enrolled inpatients was referred to as a catheter-associated UTI.

Specifically, urine samples from participants were collected in sterile containers and processed for urinalysis, but only samples with pyuria were sent for urine culture. Throughout the study, 18 urine samples were collected for analysis, including 10 samples from patients with a urinary catheter and 8 from healthy participants. Among the 10 samples from patients with an indwelling urinary catheter, 7 tested positive for pyuria and had organisms in their urine culture (UTI groups) and the remanding 3 did not (non-UTI group), as listed in Table 1. All 8 of the samples from the healthy controls tested negative for pyuria, and were also included in the non-UTI group (*n* = 7 with catheter-associated UTIs vs. *n* = 11 without UTIs). For the culture process, as a laboratory routine, 0.01 mL of urine sample was inoculated on blood and MacConkey agar (Difco Laboratories, Detroit, MI, USA) via a sterile calibrated wire loop. Bacterial growth on the media was observed after 24 h of incubation at 37 °C. For species identification, a colony subculture was performed to obtain the pure growth of the bacteria, and then was inoculated into biochemical test medias, and we observed the characteristics of the colonies to determine the species. For Gram-positive bacteria, species identification was performed by using a catalase and coagulase test [34]. Specifically, fungal isolates found in ordinary urine cultures were reported as yeast or mold, and identification of yeasts was done using a Vitek 2 YST card (bioMerieux Inc., Durham, NC, USA) [31]. All the culture procedures were performed in the microbiology laboratory at Taipei Veterans General Hospital. We confirm that all methods were performed in accordance with the relevant guidelines and regulations.

### 2.2. Measurements of Urine Samples with the ATR-FTIR Technique

The infrared spectra of the urine samples were measured with an attenuated total reflectance (ATR) technique. A Fourier-transform infrared (FTIR) spectrometer (ABB FTLA2000-104, Quebec, Quebec, Canada), a liquid-nitrogen-cooled MCT detector (Infrared Associates FTIR-16-1.0-LN2, Stuart, FL, USA), a single-bunch ATR accessory attached with a ZnSe crystal (Pike Technologies, Madison, WI, USA), and transfer optics were assembled (Mastek Technologies, Inc., XinZhuang, New Taipei City, Taiwan) to set up an ATR-FTIR spectrometer, which was used for measurement of IR data. Typically, we recorded 16–32 scans for the spectra with a resolution of 4 cm^−1^. The ZnSe crystal was cleaned with optical-grade acetone and ethanol before the measurement of each sample and a background, the spectrum of which was collected without a sample to eliminate atmospheric changes. The urine samples without any pretreatment were dropped onto the top of the ZnSe surface with a microdropper; roughly, the quantity of sample used each time was about 4 microliters. After dropping the urine sample onto the surface of the ZnSe crystal, we used a small fan to dry the sample for about 5 min to form a deposited film; meanwhile, we scanned the spectra continuously until the spectra remained constant for the dry film formed. By this means, dry films from the urine samples were prepared to avoid the strong perturbation from the liquid water in the IR region.

### 2.3. Reagents

Ethanol (C_2_H_5_OH, optical grade) and acetone (CH_3_COCH_3_, optical grade) were purchased from Merck. Urea powder (NH_2_CONH_2_), uric acid (C_5_H_4_N_4_O_3_, ≥99%), creatinine (C_4_H_7_N_3_O, ≥99.0%), and magnesium sulfate hydrate (MgSO_4_·7H_2_O, ≥98%) were purchased from Sigma-Aldrich, Saint Louis, MO, USA. Water was from a deionized source. 

## 3. Results and Discussion

### 3.1. The Culture Result and ATR-FTIR Spectra of Urine 

**Figure 1:**
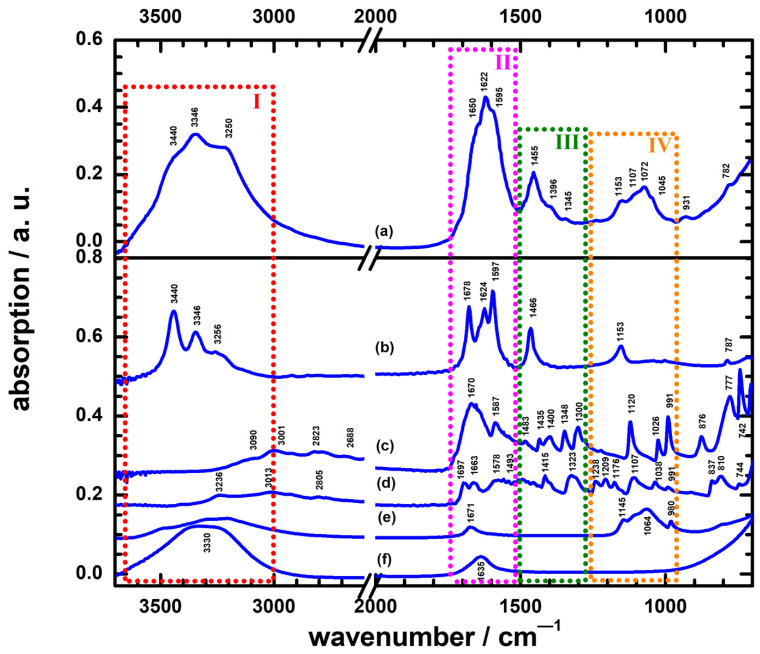
Infrared absorption spectra of: (**a**) averaged non-UTI designated as IR_av/non_; (**b**) urea; (**c**) uric acid; (**d**) creatinine; (**e**) magnesium sulfate (MgSO_4_·7H_2_O); (**f**) liquid water.

Eighteen urine samples were collected, and these samples were divided into two parts: one part was examined microscopically and cultured to identify pathogens [33,34]; the other was directly measured for their ATR-FTIR spectra. Based on the urinalysis findings, culture results, and symptoms, we concluded that 7 samples from patients with indwelling urinary catheter had pathogens causing UTI, whereas 11 samples (3 from cases with urinary catheter and 8 from healthy participants without catheter) contained no pathogen (named the non-UTI group), as listed in Table 1. Figure 1 shows the spectra of the average non-UTI; namely, IR_av/non_, and other potential species that are common in urine samples.

All non-UTIs are shown as IR_av/non_ in Figure 1a. For the purpose of comparison, Figure 1b–f also exhibit the IR absorption spectra of urea, uric acid, creatinine, magnesium sulfate (represented by MgSO_4_·7H_2_O), and liquid water, respectively; these species were expected to dominate the IR absorption spectrum of urine. Referring to the spectra in Figure 1 and previous reports [32,35], we could thus unambiguously identify the IR absorption bands of urine.

To identify the distinct bands of IR_av/non_, we specified four regions (marked in Figure 1) for interpretation. Referring to Figure 1f, a broad absorption in region I, 3650–3000 cm^−1^, for IR_av/non_ was associated with the antisymmetry stretching, symmetry stretching, and hydrogen bonds of water; after careful examination of Figure 1b, the triple pattern at 3440, 3346, and 3250 cm^−1^ on top of this broad band was determined to be due to the ν_as_ of NH, the ν_s_ of NH, and the stretching mode of OH, respectively, for urea in the urine. In region II, 1800–1500 cm^−1^, a prominent band of IR_av/non_ at 1622 cm^−1^ had shoulders at 1650 and 1595 cm^−1^; referring to Figure 1b,e, those features corresponded to absorptions at 1624, 1678, and 1597 cm^−1^ of the δ(NH_2_)/urea, ν(CO)/urea, and OH_bending_/water modes, respectively. The width of the 1622 cm^−1^ band was about 125 cm^−1^, which might have resulted from congestion from the absorption bands of 1678 cm^−1^ for urea, 1670 and 1587 cm^−1^ for uric acid, 1697 and 1663 cm^−1^ for creatinine, and 1671 cm^−1^ for MgSO_4_. In region III, 1500–1275 cm^−1^, the featured band of IR_av/non_ at 1455 cm^−1^ had a tail shoulder at 1396 cm^−1^; a weak band also appeared at 1345 cm^−1^. The absorption pattern of region III for IR_av/non_ might have arisen from absorptions of urea, uric acid, and creatinine in that region. In region IV, 1250–950 cm^−1^, a congested band of IR_av/non_ had a maximum at 1072 cm^−1^ with three shoulders at 1153, 1107, and 1045 cm^−1^; the shape of this band was evidently near that of MgSO_4_. We thus considered that the absorptions of MgSO_4_ encompassed mainly absorptions of the CN stretching modes of urea and creatinine at 1153 cm^−1^ and 1107 cm^−1^ to produce this band.

### 3.2. The Difference between the UTI and Non-UTI Groups in ATR-FTIR Spectra 

**Figure 2:**
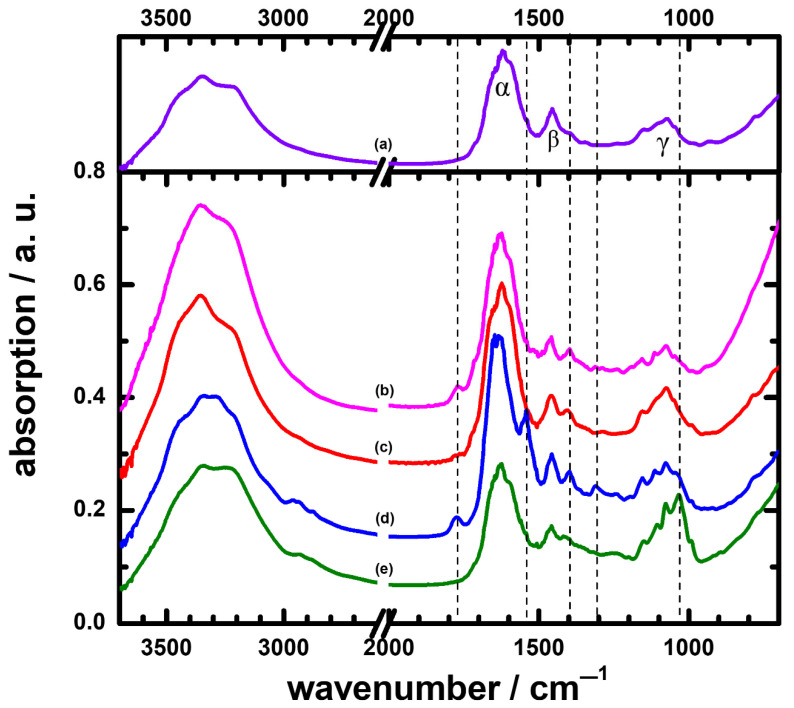
ATR-FTIR spectra of: (**a**) averaged 11 non-UTIs, designed as IR_av/non_; and UTIs cultured by yeasts: (**b**) from patient No. 2; (**c**) from patient No. 3; (**d**) from patient No. 4; (**e**) from patient No. 6.

In this work, seven urine samples from patients were identified as UTIs. According to the literature, common causative agents involved in UTIs might be *E. coli*, *P. aeruginosa*, *Staphylococcus aureus*, *Staphylococcus epidermidis*, *Klebsiella pneumoniae*, *Proteus mirabilis*, *Proteus vulgaris*, *Citrobacter freundii*, *Providentia rettgeri*, and yeast species such as *Candida albicans*, the most common yeastlike fungus [36,37]. Four UTI samples were cultured with yeast species in this work; Figure 2 plots these yeast-cultured IR spectra of the UTIs, with the IR_av/non_ used for comparison. Although the number of patients was small, the assignments were also included for the reference in the differential diagnosis in patients. The corresponding shoulders in the IR spectra of yeast at ~2925 cm^−1^ and ~2875 cm^−1^ had contributions of CH_2_ stretching vibrations of CH_2_OH groups in lipids. The bands just below 1650 cm^−1^ and around 1560 cm^−1^ are called amide I and amide II, respectively, and reflect the presence of chitin, a component of the yeast cell wall, and probably some products of protein degradation [38]. The IR features at 1456 cm^−1^ and 1250 cm^−1^ indicated the CH_3_ asymmetry stretching and amide III of protein, respectively. The high absorptions <1200 cm^−1^ were characteristic of polysaccharides in the yeast cells. The bands at 1157 cm^−1^ and 1080 cm^−1^ were mainly due to CO stretching of the carbohydrates and PO_2_ of the nucleic acids, respectively [39].

**Figure 3:**
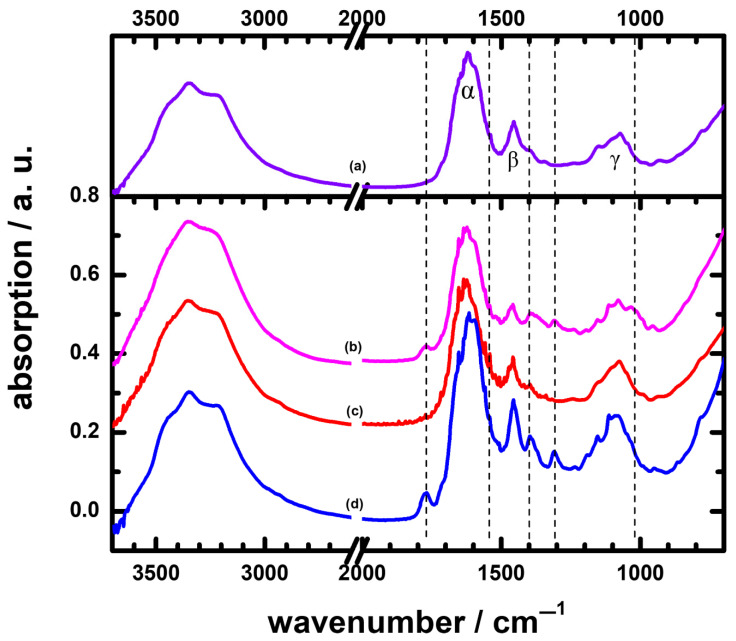
ATR-FTIR spectra of: (**a**) averaged 11 non-UTIs, assigned as IR_av/non_; and UTI group and cultured bacteria: (**b**) *E. faecium* (patient No. 1); (**c**) *E. coli* (patient No. 5); (**d**) *P. aeruginosa* (patient No. 7).

The ATR-IR spectrum of urine cultured with *E. faecium* is shown in Figure 3b. The prominent bands around 1618 cm^−1^ and 1456 cm^−1^ were assigned to the NH bending and CN stretching modes of urea in the urine. The amide I had an absorption ~1650 cm^−1^. The peak in the region of 1600–1540 cm^−1^ also overlapped the amide II of bacteria. The carboxylic groups of bacterial cells exhibited both distinct bands at ∼1720 cm^−1^ and 1394 cm^−1^, respectively, corresponding to C=O and symmetric stretching of COO^−^. The bands at 1240 cm^−1^ and 1114 cm^−1^ indicated PO_2_ asymmetric stretching of nucleic acids and phospholipids of teichoic acid cell walls [40]. Finally, the spectral range of 1200 to 900 cm^−1^ covered the region of P=O symmetric stretching and symmetrical CO–O–C stretching vibrations of carbohydrates due to the presence of peptidoglycans in cell walls.

As for the urine cultured with *E. coli,* the IR spectrum is presented in Figure 3c. The absorption of smaller broad bands at 2985–2875 cm^−1^ resulted from the C–H asymmetry and symmetry stretching of CH_3_ and CH_2_ in fatty acids. The large bands in the raw spectrum at 1755–1500 cm^−1^ were associated with the merging of the amide I and amide II groups of proteins [41,42]. The band at approximately 1396 cm^−1^ was caused by the vibration of C=O symmetric stretching of the COO^−^ group in amino acids and fatty acids. The smaller band at 1243 cm^−1^ and the slightly larger band at 1045 cm^−1^ corresponded to the P=O asymmetry stretching and symmetry stretching in phospholipids, respectively.

Figure 3d shows an IR spectrum of urine cultured with *P. aeruginosa*. The bands in the region of 3000–2800 cm^−1^ were CH vibrational modes in CH_3_ and >CH_2_ asymmetry and symmetry stretching modes of fatty acids and lipids. The amide I band of proteins was at 1650 cm^−1^, and the amide II band of C-N stretching and N-H bending in proteins and peptides was at 1560 cm^−1^. The spectral bands at 1456, 1398, and 1309 cm^−1^ received a contribution from C–H bending vibrations of CH_2_, >C–O bending from carboxylic acids, and >P=O symmetric stretching, corresponding to fatty acids, proteins, and phosphorus-containing carbohydrates, respectively. The composite bands around ~1080 cm^−1^ were due to cell carbohydrate polymers [43,44,45].

After justifying the spectra in Figure 2, we assessed differences in spectra among the urine samples in the wavenumber region 1800–800 cm^−1^. In particular, the shapes of absorptions in region IV, 1250–950 cm^−1^, appeared quite obviously different for the yeast-cultured UTIs and IR_av/non_; this indicated that we might discriminate the UTIs from the non-UTIs by comparing their IR absorption curves in the 1250–950 cm^−1^ region (as shown in Figures S1 and S2 in the Supplementary Materials). Unless a quantitative method is developed, this scheme was rather a qualitative identification.

### 3.3. The Characterization Method in ATR-FTIR Spectra to Discriminate Non-UTIs and UTIs with Yeast Species

For the purpose of recognition of UTIs from urine samples in a more precise manner, we further assigned the bands near 1625, 1456, and 1075 cm^−1^ as α, β, and γ bands, which are also marked in Figure 2 for clarity. Upon careful examination, we found that the ratio of their intensities could serve as an indicator to distinguish UTIs from non-UTIs. For example, the ratio of α, β, and γ for the IR_av/non_ was R_α(av/non)_:R_β(av/non)_:R_γ(av/non)_ = 100:45:30, whereas those of the yeast-cultured spectra were R_α2_:R_β2_:R_γ2_ = 100:35:21, R_α3_:R_β3_:R_γ3_ = 100:33:30, R_α4_:R_β4_:R_γ4_ = 100:37:28, and R_α6_:R_β6_:R_γ6_ = 100:44:53 for patient Nos. 2, 3, 4, and 6, respectively. We noticed that the absolute deviation of the value for either R_βn_ or R_γn_ was greater than 7 from values R_β(av/non)_ = 45 or R_γ(av/non)_ = 30; the urine sample was then cultured with yeast species. The values of R_αn_, R_βn_, and R_γn_ are listed in Table 2. By this means, a calculation of the deviation value of R_βn_ or R_γn_ in the IR spectrum was a more quantitative way for identification of UTIs. 

### 3.4. The Characterization Method in ATR-FTIR Spectra to Discriminate Different Pathogens in UTI Groups

Figure 3 displays the ATR-FTIR spectra of the urine samples cultured with bacterial types *E. faecium*, *E. coli*, and *P. aeruginosa*. The values of R_αn_, R_βn_, and R_γn_ for these samples are also listed in Table 2. Although the number of cases was relatively small in this pilot study, the characteristic method for different pathogens in the UTI groups was proposed as a clue for researchers in a future study. Notably, the values of R_β1_, R_β5_, and R_β7_ were −12, −9 and +8, the absolute values for which were greater than 7. According to this scheme, these urine samples belonged to UTIs; the results were consistent with the outcome from cultures. Generally, the differences in intensities for these bands might be related to the cell-wall structures of Gram-positive, Gram-negative, and yeast species. 

Again, the shapes of absorptions in region IV, 1250–950 cm^−1^, for bacterial types of UTIs also appeared obviously dissimilar to that of the IR_av/non_; we might use the IR absorption shapes in region 1250–950 cm^−1^ to discriminate the UTIs from the non-UTIs. One possible method of analysis, using differences in the absorption shapes in this region, might be distinguished by comparisons of the normalized second derivative spectra of the UTIs with that of the non-UTIs, as displayed in the Supplementary Materials (Figure S1). 

Remarkably, new IR bands appeared in the spectra of UTIs; the positions of these bands are marked with dashed lines in Figure 2 and Figure 3 and are also listed in Table 2. These bands might be characteristic of a UTI; for example, the bands near 1395–1410 cm^−1^ for patient Nos. 1, 2, 3, 4, 5, and 7 were due to C-H bending vibrations of CH_2_ and COO^−^ symmetric stretching of proteins [32,43] of the causative agents involved in UTIs, as well as to the elevated leukocyte counts in the urine, an indicator of inflammation (Table 2). Notably, urine sample Nos. 4, 6, and 7, with relatively higher leukocyte counts, had higher IR intensities than the others. An observation of a new band in the IR of urine, as listed in Table 2, can thus mean a case of a UTI in a patient. At present, we have inadequate information to demonstrate a causative link between these new bands and UTIs. To understand the formation of these bands, we suggest undertaking a thorough investigation in future. For example, the new band near 1770 cm^−1^ might be associated with the symmetrical C=O stretching vibration of an anhydride. There is great interest in exploring how an anhydride compound is involved in the development of UTIs; the mechanism of formation is also intriguing to investigate. Those topics were, however, beyond the scope of the present work.

The goal of this work was to test the feasibility of using molecular spectra as a diagnostic tool. We selected the carrier from the urine and diagnosed the UTI from the IR spectra. As demonstrated in Figure 1, Figure 2 and Figure 3, Figures S1 and S2, and as summarized in data in Table 2, we obtained positive correlations for diagnosis of UTIs from IR absorption spectra of the urine samples. First of all, UTIs might result in new IR absorption bands, which could be detected and serve as clues for UTIs. In addition, the values of the ratios of intensities for the bands near 1625 cm^−1^ (α), 1456 cm^−1^ (β), and 1075 cm^−1^ (γ) from the UTIs deviated from those of normal values from the non-UTIs; by this means, those values could aid in the identification of the infections. 

Further, the spectral profiles between the UTIs and non-UTIs revealed variations in the wavenumber region of 1250–950 cm^−1^; this diversity in the spectral profiles provided additional confirmation of the infections. As shown in Figure S1, the spectra of UTIs in patients’ urine caused by different pathogens and non-UTIs were stacked by comparison of the second derivative. Several spectra differences at 1216, 1176, 1134, 1097, and 973 cm^−1^ for *E. faec.*; 1130, 1092, 1024, and 960 cm^−1^ for *E.* *coli*; 1174, 1095, 1051, 1008, and 970 cm^−1^ for *P. aerug.*; and 1215, 1180, 1005, and 972 cm^−1^ for yeast were also found as potential discriminative wavenumbers. 

Although there was no further discussion of the profiles in region 1250–950 cm^−1^, we believe that the analytical methods of principal component analysis (PCA) or principal regression analysis (PRA) can be used to recognize infections in this region. For demonstration purposes, we also derived the variations in the spectral profiles based on an analysis of the area for this band, as shown in Figure S2 and listed in Table S1 in the Supplementary Materials. Regarding the correlation between the high intensity found at 1395–1410 cm^−1^ and the urinary leukocyte response, it warrants large-scale studies to evaluate whether any other intensity region was associated with the severity of pyuria.

Although the tests of the assay were performed using real urine samples from the patients, the small number of patients remains one of major limitations of the pilot study. Thus, this warrants a large-scale study with an increased number of urine specimens from more patients or simulated experiments using artificially infected urine samples with different bacteria to validate the findings. In addition, since the concentrations of yeast in four urine samples were the same and relatively high (all reported as >10^5^ colony-forming units/mL, data not shown in the Results section) and the other three samples were infected by three different bacteria species, it was difficult for us to assess the association between the bacterial load and the intensity of the IR bands of specific pathogens. In view of further clinical application, this also warrants study to assess the limit of detection of the proposed FTIR method by using more urine samples with bacterial loads ranging from relatively low to high. Although not many urine samples were tested in this work, the results were encouraging for diagnostic purposes. A successful development of this technique for the diagnosis of diseases has advantages over general diagnostics. Essentially, time can be saved, with less than 10 min required for this ATR-FTIR method using urine for the confirmation of UTIs; this advantage will much improve the medical treatment of patients. From a technical point of view, this diagnostic method using IR spectra has a potential ability to become developed as an automatic scheme for diagnosis; importantly, applying this technique will save labor in a hospital. Incidentally, although we used the ATR technique in this work, other sampling methods are also applicable for IR spectra, including the transmission mode and photoacoustic detection method. Moreover, we proposed a calculation method in ATR-IR to discriminate UTIs from non-UTIs, as well as different pathogens associated with UTIs. 

## 4. Conclusions

To test the feasibility of using IR spectra for diagnostic purposes, we recorded the IR spectra of urine samples with the ATR technique, and focused on the diagnosis of UTIs in patients. By this means, we recorded the ATR-FTIR spectra of 18 urine samples, including 11 non-UTIs and 7 UTIs. We averaged the IR spectra of 11 non-UTIs as a standard non-UTI spectrum (IR_av/non_), and found that IR spectra of each UTI exhibited its characteristic new bands, which could serve as clues for infections. Notably, the ratio values of the intensities of bands near 1625 cm^−1^ (α), 1456 cm^−1^ (β), and 1075 cm^−1^ (γ) for the UTIs differed from those for non-UTIs. Moreover, in the UTI samples, the spectral profiles in the wavenumber region of 1250–950 cm^−1^ presented a specific pattern for different pathogens. Several spectra differences at 1216, 1176, 1134, 1097, and 973 cm^−1^ for *E. faec.*; 1130, 1092, 1024, and 960 cm^−1^ for *E.* *coli*; 1174, 1095, 1051, 1008, and 970 cm^−1^ for *P. aerug.*; and 1215, 1180, 1005, and 972 cm^−1^ for yeast were also found as potential discriminative wavenumbers to distinguish different pathogen infections. Furthermore, a high IR intensity region at 1395–1410 cm^−1^ was correlated with the elevated leukocyte counts in the urine. As a pilot study, our findings suggested that this calculation method using ATR-FTIR may provide clues to detect UTIs and other diseases in the future.

## Figures and Tables

**Table 1:** Clinical characteristics of the urine samples (*n* = 18).

Diagnosis	Group	Culture Result	Type
Non-UTI (*n* = 11)	1	No growth (*n* = 11)	
UTI (*n* = 7)	2	*E. coli* (*n* = 1)	GN bacterium
	3	Yeast (*n* = 4)	Fungi
	4	*P. aeruginosa* (*n* = 1)	GN bacterium
	5	*E. faecium* (*n* =1)	GP bacterium

GN: Gram-negative; GP: Gram-positive.

**Table 2:** Comparison of ratios of intensities for bands near 1625 cm^−1^ (α), 1456 cm^−1^ (β), and 1075 cm^−1^ (γ), and new bands observed in ATR-FTIR spectra of UTI.

	R_α_:R_β_:R_γ_ ^(a)^	ΔR_α_:ΔR_β_:ΔR_γ_	New Bands/cm^−1^	WBC/HPF ^(b)^
IR_av/non_	100:45:30	0:0:0	-	<5
No. 1 (*E. faec.*)	100:33:28	0:−12:−2	1771, 1396, 1310	6–10
No. 2 (*yeast*)	100:35:21	0:−10:−9	1772, 1398	20–29
No. 3 (*yeast*)	100:33:30	0:−12:0	1771, 1405	20–29
No. 4 (*yeast*)	100:37:28	0:−8:−2	1774, 1541, 1398, 1312	>100
No. 5 (*E. coli*)	100:36:31	0:−9:+1	1396	20–29
No. 6 (*yeast*)	100:44:53	0:−1:+23	1033, 992	50–99
No. 7 (*P. aerug.*)	100:53:35	0:+8:+5	1772, 1397, 1311	>100

^(a)^ The intensity of the band near 1625 cm^−1^ (α) was assigned a value of 100; the ratio values of bands near 1456 cm^−1^ (β) and 1075 cm^−1^ (γ) were then calculated from their intensities relative to the band near 1625 cm^−1^ (α). ^(^^b)^ WBC/HPF: white blood cell (leukocyte)/high-power field.

## Supplementary Materials

The following supporting information can be downloaded at: https://www.mdpi.com/article/10.3390/s22103638/s1, S1. Second derivation of the spectral profiles between the UTIs and non-UTIs in wavenumber region 950–1250 cm^−1^, including Figure S1; S2. Derivation of the variations of the spectral profiles between the UTIs and non-UTIs in wavenumber region 950–1250 cm^−1^, including Figure S2 and Table S1.

## References

[1] Simmering J.E., Tang F., Cavanaugh J.E., Polgreen L.A., Polgreen P.M. (2017). The Increase in Hospitalizations for Urinary Tract Infections and the Associated Costs in the United States, 1998–2011. Open Forum Infect. Dis..

[2] Davenport M., Mach K.E., Shortliffe L.M.D., Banaei N., Wang T.H., Liao J.C. (2017). New and developing diagnostic technologies for urinary tract infection. Nat. Rev. Urol..

[3] Simerville J.A., Maxted W.C., Pahira J.J. (2005). Urinalysis: A comprehensive review. Am. Fam. Phys..

[4] Navarro D.F., Sullivan F., Azcoaga-Lorenzo A., Santiago V.H. (2020). Point-of-care tests for urinary tract infections: Protocol for a systematic review and meta-analysis of diagnostic test accuracy. BMJ Open.

[5] Kapur S., Gehani M., Kammili N., Bhardwaj P., Nag V., Devara S.M., Sharad S. (2019). Clinical validation of innovative optical-sensor-based, low-cost, rapid diagnostic test to reduce antimicrobial resistance. J. Clin. Med..

[6] Kotsilkov K., Popova C., Boyanova L., Setchanova L., Mitov I. (2015). Comparison of culture method and real-time PCR for detection of putative periodontopathogenic bacteria in deep periodontal pockets. Biotechnol. Equip..

[7] Goris J., Konstantinidis K.T., Klappenbach J.A., Coenye T., Vandamme P., Tiedje J.M. (2007). DNA–DNA hybridization values and their relationship to whole-genome sequence similarities. Int. J. Syst. Evol. Microbiol..

[8] An J., Jiang Y., Shi B., Wu D., Wu W. (2020). Low-cost battery-powered and user-friendly real-time quantitative PCR system for the detection of multigene. Micromachines.

[9] Bangaoil R., Santillan A., Angeles L.M., Abanilla L., Lim A., Ramos M.C., Fellizar A., Guevarra L., Albano P.M. (2020). ATR-FTIR spectroscopy as adjunct method to the microscopic examination of hematoxylin and eosin-stained tissues in diagnosing lung cancer. PLoS ONE.

[10] Sala A., Anderson D.J., Brennan P.M., Butler H.J., Cameron J.M., Jenkinson M.D., Rinaldi C., Theakstone A.G., Baker M.J. (2020). Biofluid diagnostics by FTIR spectroscopy: A platform technology for cancer detection. Cancer Lett..

[11] Ho C.S., Jean N., Hogan C.A., Blackmon L., Jeffrey S.S., Holodniy M., Banaei N., Saleh A.A.E., Ermon S., Dionne J. (2019). Rapid identification of pathogenic bacteria using Raman spectroscopy and deep learning. Nat. Commun..

[12] Zhang W., He S., Hong W., Wang P. (2022). A Review of Raman-Based Technologies for Bacterial Identification and Antimicrobial Susceptibility Testing. Photonics.

[13] Rousseau A.N., Faure N., Rol F., Sedaghat Z., Le Galudec J., Mallard F., Josso Q. (2021). Fast Antibiotic Susceptibility Testing via Raman Microspectrometry on Single Bacteria: An MRSA Case Study. ACS Omega.

[14] Ciloglu F.U., Caliskan A., Saridag A.M., Kilic I.H., Tokmakci M., Kahraman M., Aydin O. (2021). Drug-resistant Staphylococcus aureus bacteria detection by combining surface-enhanced Raman spectroscopy (SERS) and deep learning techniques. Sci. Rep..

[15] Tahir M.A., Dina N.E., Cheng H., Valev V.K., Zhang L. (2021). Surface-enhanced Raman spectroscopy for bioanalysis and diagnosis. Nanoscale.

[16] Patel R. (2015). MALDI-TOF MS for the Diagnosis of Infectious Diseases. Clin. Chem..

[17] Qiu G., Gai Z., Saleh L., Tang J., Gui T., Kullak-Ublick G.A., Wang J. (2021). Thermoplasmonic-Assisted Cyclic Cleavage Amplification for Self-Validating Plasmonic Detection of SARS-CoV-2. ACS Nano.

[18] Gür S.D., Bakhshpour M., Denizli A. (2019). Selective detection of Escherichia coli caused UTIs with surface imprinted plasmonic nanoscale sensor. Mater. Sci. Eng. C.

[19] Farraia M.V., Rufo J.C., Paciência I., Mendes F., Delgado L., Moreira A. (2019). The electronic nose technology in clinical diagnosis: A systematic review. Porto Biomed. J..

[20] Flores-Gonzalez J., Cancino-Díaz J.C., Chavez-Galan L. (2020). Flow Cytometry: From Experimental Design to Its Application in the Diagnosis and Monitoring of Respiratory Diseases. Int. J. Mol. Sci..

[21] Stobiecka M., Dworakowska B., Jakiela S., Lukasiak A., Chalupa A., Zembrzycki K. (2016). Sensing of survivin mRNA in malignant astrocytes using graphene oxide nanocarrier-supported oligonucleotide molecular beacons. Sens. Actuators B Chem..

[22] Ratajczak K., Krazinski B.E., Kowalczyk A.E., Dworakowska B., Jakiela S., Stobiecka M. (2018). Hairpin-Hairpin Molecular Beacon Interactions for Detection of Survivin mRNA in Malignant SW480 Cells. ACS Appl. Mater. Interfaces.

[23] Ratajczak K., Stobiecka M. (2020). High-performance modified cellulose paper-based biosensors for medical diagnostics and early cancer screening: A concise review. Carbohydr. Polym..

[24] Stobiecka M., Ratajczak K., Jakiela S. (2019). Toward early cancer detection: Focus on biosensing systems and biosensors for an anti-apoptotic protein survivin and survivin mRNA. Biosens. Bioelectron..

[25] Santos M.C.D., Morais C.L.M., Lima K.M.G. (2020). ATR-FTIR spectroscopy for virus identification: A powerful alternative. Biomed. Spectrosc. Imaging.

[26] Sharma A., Agrawal A., Awasthi K.K., Awasthi K., Awasthi A. (2021). Biosensors for diagnosis of urinary tract infections: Advances and future challenges. Mater. Lett. X.

[27] Qiu G., Ng S.P., Lawrence Wu C.M. (2015). Differential phase-detecting localized surface plasmon resonance sensor with self-assembly gold nano-islands. Opt. Lett..

[28] Heise H.M., Voigt G., Lampen P., Küpper L., Rudloff S., Werner G. (2001). Multivariate calibration for the determination of analytes in urine using mid-infrared attenuated total reflection spectroscopy. Appl. Spectrosc..

[29] Flores-Mireles A., Hreha T.N., Hunstad D.A. (2019). Pathophysiology, treatment, and prevention of catheter-associated urinary tract infection. Top. Spinal Cord Inj. Rehabil..

[30] Flores-Mireles A.L., Walker J.N., Caparon M., Hultgren S.J. (2015). Urinary tract infections: Epidemiology, mechanisms of infection and treatment options. Nat. Rev. Microbiol..

[31] Yang S.-P., Chen Y.-Y., Hsu H.-S., Wang F.-D., Chen L.-Y., Fung C.-P. (2013). A risk factor analysis of healthcare-associated fungal infections in an intensive care unit: A retrospective cohort study. BMC Infect. Dis..

[32] Steenbeke M., Bruyne S.D., Boelens J., Oyaert M., Glorieux G., Biesen W.V., Linjala J., Delanghe J.R., Speeckaert M.M. (2020). Exploring the possibilities of infrared spectroscopy for urine sediment examination and detection of pathogenic bacteria in urinary tract infections. Clin. Chem. Lab. Med..

[33] Ho M.-L., Liu W.-F., Tseng H.-Y., Yeh Y.-T., Tseng W.-T., Chou Y.-Y., Huang X.-R., Hsu H.-C., Ho L.-I., Pan S.-W. (2020). Quantitative determination of leukocyte esterase with a paper-based device. RSC Adv..

[34] Cheesbrough M. (2005). District Laboratory Practice in Tropical Countries, Part 2.

[35] Takamura A., Watanabe K., Akutsu T., Ozawa T. (2018). Soft and robust identification of body fluid using Fourier transform infrared spectroscopy and chemometric strategies for forensic analysis. Sci. Rep..

[36] Nicolle L.E. (2005). Catheter-related urinary tract infection. Drugs Aging.

[37] Chen L.F., Ou T.Y., Teng S.O., Chen F.-L., Hsieh T.C., Lee W.S. (2014). Hospital-acquired urinary tract infections in patients with diabetes and urinary catheterization. J. Exp. Clin. Med..

[38] Novák M., Synytsya A., Gedeon O., Slepička P., Procházka V., Synytsya A., Blahovec J., Hejlová A., Čopίková J. (2012). Yeast ß(1-3),(1-6)-D-glucan films: Preparation and characterization of some structural and physical properties. Carbohydr. Polym..

[39] Salman A., Tsror L., Pomerantz A., Moreh R., Mordechai R., Huleihel M. (2010). FTIR spectroscopy for detection and identification of fungal phytopathogenes. Spectroscopy.

[40] Nitosetein T., Wongwattanakul M., Chonanant C., Leelayuwat C., Charoensri N., Jearanaikoon P., Lulitanond A., Wood B.R., Tippayawat P., Heraud P. (2021). Attenuated Total Reflection Fourier Transform Infrared Spectroscopy combined with chemometric modelling for the classification of clinically relevant Enterococci. J. Appl. Microbiol..

[41] Sharaha U., Rodriguez-Diaz E., Sagi O., Riesenberg K., Salman A., Bigio I.J., Huleihel M. (2019). Fast and reliable determination of Escherichia coli susceptibility to antibiotics: Infrared microscopy in tandem with machine learning algorithms. J. Biophotonics.

[42] Lee J., Ahn M.S., Lee Y.-L., Jie E.Y., Kim S.-G., Kim S.W. (2018). Rapid tool for identification of bacterial strains using Fourier transform infrared spectroscopy on genomic DNA. J. Appl. Microbiol..

[43] Soler-Arango J., Figoli C., Muraca G., Bosch A., Brelles-Mariño G. (2019). The Pseudomonas aeruginosa biofilm matrix and cells are drastically impacted by gas discharge plasma treatment: A comprehensive model explaining plasmamediated biofilm eradication. PLoS ONE.

[44] McWhirter M.J., McQuillan A.J., Bremer P.J. (2002). Influence of ionic strength and pH on the first 60 min of *Pseudomonas aeruginosa* attachment to ZnSe and to TiO_2_ monitored by ATR-IR spectroscopy. Colloids Surf. B.

[45] Martak D., Valot B., Sauget M., Cholley P., Thouverez M., Bertrand X., Hocquet D. (2019). Fourier-Transform InfraRed Spectroscopy Can Quickly Type Gram-Negative Bacilli Responsible for Hospital Outbreaks. Front. Microbiol..

