# Supplementary material for: Using an ATR-FTIR Technique to Detect Pathogens in Patients with Urinary Tract Infections: A Pilot Study
Source: Sensors (Basel). 2022 May 10;22(10):3638. doi: 10.3390/s22103638 (PMC9147530; doi:10.3390/s22103638)
Supplement: Supplementary file 1 [file sensors-22-03638-s001.zip › sensors-1694090-supplementary.pdf]

sensors

Article

Using an ATR-FTIR Technique to Detect Pathogens in Patients with Urinary Tract Infections: A Pilot Study

Sheng-Wei Pan [1,2,3,†], Hsiao-Chi Lu [4,†], Jen-Iu Lo [4], Li-Ing Ho [1], Ton-Rong Tseng [5], Mei-Lin Ho [6,*] and Bing-Ming Cheng [4,7,*]

[1] Department of Chest Medicine, Taipei Veterans General Hospital, Taipei 11217, Taiwan; swpan2@vghtpe.gov.tw (S.-W.P.); liho@vghtpe.gov.tw (L.-I.H.)
[2] School of Medicine, National Yang Ming Chiao Tung University, Taipei 12304, Taiwan
[3] Division of Pulmonary, Critical Care and Sleep Medicine, Department of Medicine, University of California San Diego, La Jolla, CA 92093, USA
[4] Department of Medical Research, Hualien Tzu Chi Hospital, Buddhist Tzu Chi Medical Foundation, No. 707, Sec. 3, Chung-Yang Rd., Hualien City 97002, Taiwan; hchopelu@gmail.com (H.-C.L.); jeniuluo@gmail.com (J.-I.L.)
[5] Mastek Technologies, Inc. 4F-4, No. 13, Wuquan 1st Rd., Xinzhuang, New Taipei City 24892, Taiwan; simon@mastek-tw.com
[6] Department of Chemistry, Soochow University, No. 70, LinShih Rd., Shih-Lin, Taipei 11102, Taiwan
[7] Office of Research and Development, Tzu Chi University of Science and Technology, No. 880, Sec. 2, Chien-kuo Rd., Hualien City 97005, Taiwan
* Correspondence: meilin_ho@gm.scu.edu.tw (M.-L.H.); bmcheng7323@gmail.com (B.-M.C.); Tel.: +886-2-28819471 (ext. 6827) (M.-L.H.)
† These authors contributed equally to this work.

Citation: Pan, S.-W.; Lu, H.-C.; Lo, J.-I.; Ho, L.-I.; Tseng, T.-R.; Ho, M.-L.; Cheng, B.-M. Using an ATR-FTIR Technique to Detect Pathogens in Patients with Urinary Tract Infections: A Pilot Study. *Sensors* **2022**, *22*, 3630. https://doi.org/10.3390/s22103630

Academic Editors: Wenfeng Zheng, Yichao Yang, Chao Liu and Wenshuo Zhou

Received: 7 April 2022
Accepted: 7 May 2022
Published: 10 May 2022

Publisher's Note: MDPI stays neutral with regard to jurisdictional claims in published maps and institutional affiliations.

Copyright: © 2022 by the authors. Licensee MDPI, Basel, Switzerland. This article is an open access article distributed under the terms and conditions of the Creative Commons Attribution (CC BY) license (https://creativecommons.org/licenses/by/4.0/).

S1. Second derivation of the spectral profiles between the UTI and non-UTI in wave-number region 950–1250 cm^{-1}

For analysis, the zero-offset position of spectrum was taken at 4000 cm^{-1}; then, the spectrum was normalized at the 1622 cm^{-1}. Subsequently, the spectrum was performed an analysis using a smoothing model with second-order derivative, 2 for polynomial order and 20 for points of window. Second differentiation spectra from the original were carried out using the Savitzky–Golay method in Origin 8.5 software. Afterward, the second derivative of IR spectrum was calculated and used for representing alterations in the rate of change of absorbance ($d^2A/d\upsilon^2$). By this means, each band represented in the second derivative for urine from UTI displays a more pronounced minimum than that from non-UTI, as shown in Figure S1.

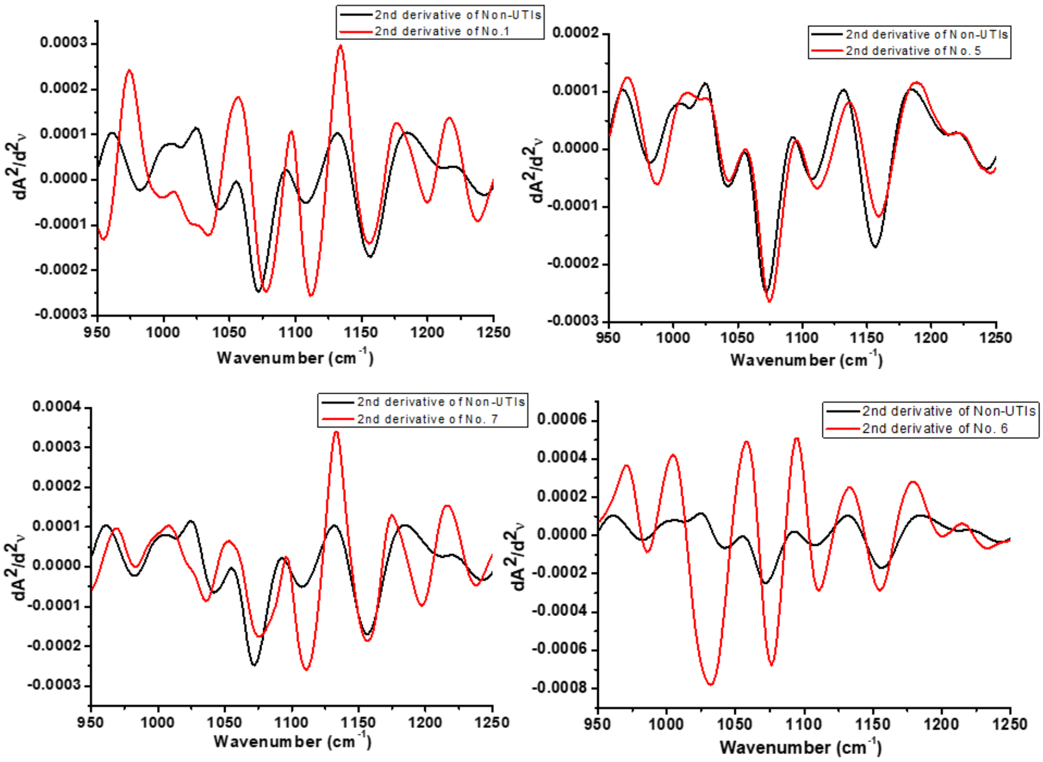

Figure S1. Comparison of the averaged second derivatives of the spectra for urines from UTIs caused by different pathogens (Patients No. 1, 5, 7, 6; red) and Non-UTI group (black).

S2. Derivation of the variations of the spectral profiles between the UTI and non-UTI in wavenumber region 950–1250 cm^{-1}

The spectral profiles between the UTI and non-UTI reveal variations in wavenumber region 950–1250 cm^{-1}; this diversity in the spectral profiles provides additional confirmation of the infections. We derived these variations based on the integrated area of the bands in region 950–1250 cm^{-1}. For this purpose, we offset the spectra by setting the absorption in the wavenumber range 1900–1950 cm^{-1} as zero for the first step; subsequently, we integrated the area of absorptions in the wavenumber range 890–1950 cm^{-1} as normalization factors. Finally, the absolute variations of area in the IR range 950–1250 cm^{-1} of UTI patients compared to that of the averaged non-UTI spectrum $IR_{av/non}$ were obtained. For demonstration, the diagram for derivation of absolute variations from Figure 1 is shown in Figure S2; those values are listed in the Table S1.

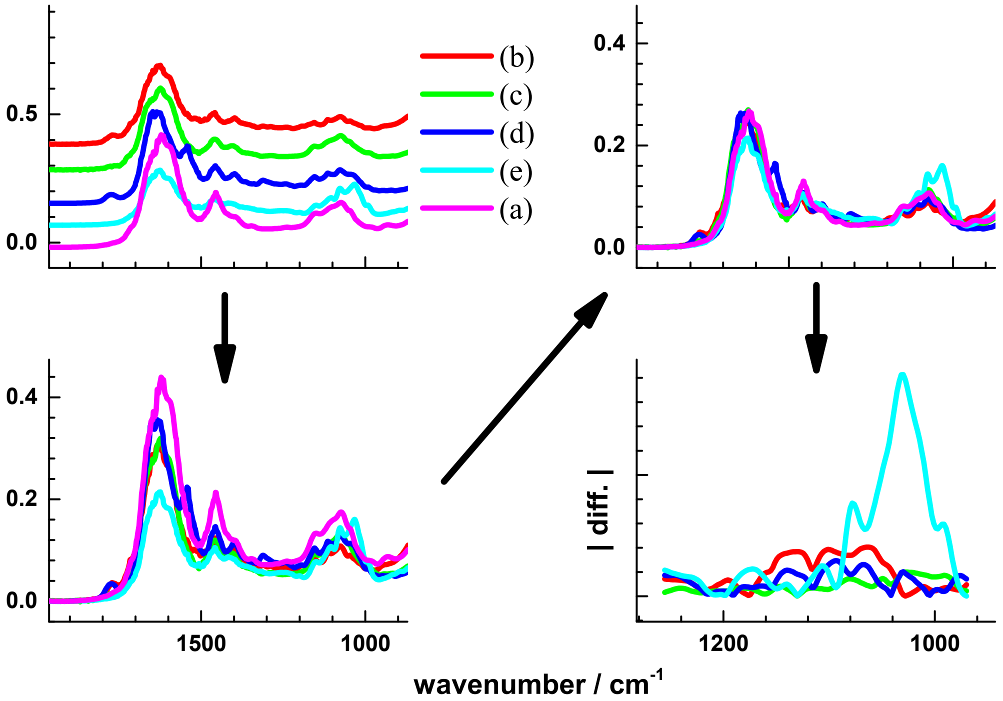

Figure S2. The diagram for derivation of absolute variations from Figure 1.

Table S1. The absolute variations of area in the IR range 950–1250 cm^{-1} of UTI patients compared to that of the averaged non-UTI spectrum IRav/non.

**Patient**	**Spectrum**	**Absolute Variations (%)**
No. 1	Figure 3b	12.3
No. 2	Figure 2b	14.5
No. 3	Figure 2c	6.1
No. 4	Figure 2d	9.6
No. 5	Figure 3c	6.1
No. 6	Figure 2e	24.4
No. 7	Figure 3d	9.9
